# Identifying the physical features of marina infrastructure associated with the presence of non-native species in the UK

**DOI:** 10.1007/s00227-016-2941-8

**Published:** 2016-07-25

**Authors:** Victoria Foster, Rebecca J. Giesler, A. Meriwether W. Wilson, Christopher R. Nall, Elizabeth J. Cook

**Affiliations:** 1 Scottish Association for Marine Science, Scottish Marine Institute, Oban, Argyll PA37 1QA UK; 2 School of GeoSciences, University of Edinburgh, Edinburgh, EH93JW Scotland UK; 3 Environmental Research Institute, University of Highlands and Islands, Thurso, Caithness KW14 7EE UK

## Abstract

Marine invasive non-native species (NNS) are one of the greatest threats to global marine biodiversity, causing significant economic and social impacts. Marinas are increasingly recognised as key reservoirs for invasive NNS. They provide submersed artificial habitat that unintentionally supports the establishment of NNS introduced from visiting recreational vessels. While ballast water and shipping vectors have been well documented, the role of recreational vessels in spreading NNS has been relatively poorly studied. Identification of the main physical features found within marinas, which relate to the presence of NNS, is important to inform the development of effective biosecurity measures and prevent further spread. Towards this aim, physical features that could influence the presence of NNS were assessed for marinas throughout the UK in July 2013. Thirty-three marine and brackish NNS have been recorded in UK marinas, and of the 88 marinas studied in detail, 83 contained between 1 and 13 NNS. Significant differences in freshwater input, marina entrance width and seawall length were associated with the presence of NNS. Additionally, questionnaires were distributed to marina managers and recreational vessel owners to understand current biosecurity practices and attitudes to recreational vessel biosecurity. The main barriers to biosecurity compliance were cited as cost and time. Further work identifying easily distinguished features of marinas could be used as a proxy to assess risk of invasion.

**Electronic supplementary material:**

The online version of this article (doi:10.1007/s00227-016-2941-8) contains supplementary material, which is available to authorized users.

## Introduction

Biological invasions by non-native species (NNS) are generally accepted to be one of the greatest threats to biodiversity worldwide (CBD 1992). Invasive NNS can cause significant economic and social impacts and are estimated to cause global damage amounting to $120 billion annually (Pimentel et al. 2005). Biosecurity measures including quarantine, customs and legislative controls have long been in place for terrestrial pest species (Cook et al. 2016). During the last decade, marine invasive NNS have received increased attention from both scientists and policy makers (Genovesi et al. 2014; Hulme 2009, 2015); yet both ecological knowledge and biosecurity practices to address marine invasive NNS still lag behind those of terrestrial species (Williams and Grosholz 2008; Cook et al. 2016).

To date, commercial vessels have been the primary focus for marine NNS biosecurity measures, due to long voyages across biogeographic boundaries and the ability of organisms to be transported by ballast water (Briski et al. 2013; Seebens et al. 2013). Recreational vessels were generally considered low-risk vectors due to shorter voyages, frequent cleaning regimes and high home port fidelity (Ashton et al. 2006a). However, recreational boating has now been identified as a significant vector for the introduction and spread of NNS, especially at more local scales, allowing the secondary spread of these species away from sites of first introduction (Boos et al. 2011; Clarke Murray et al. 2011; Ashton et al. 2014; Zabin et al. 2014). Recreational vessels can transport invasive NNS via hull fouling, internal fouling in pipes, in ballast, bilge or anchor-well water, and in inlets leading off the hull (Darbyson et al. 2009; Acosta and Forrest 2009). The short, relatively slow voyages typical of recreational vessels make successful spread more likely, as there is a higher probability that fouling species will survive (Coutts et al. 2010; Clarke Murray et al. 2011). Furthermore, they are more likely to be able to colonise the receiving site, given that the environmental and climatic conditions are likely to be similar to the source habitat (Minchin 2006; Coutts et al. 2010).

Measures to control marine invasive NNS once they have become established can be costly and time-consuming (Hulme 2009). It is, therefore, key to target high-risk entry points and manage the critical pathways in order to eradicate the initial introduction before species are able to establish and spread (Katsanevakis et al. 2013; Williams et al. 2013). Control of the secondary spread of established marine NNS is also crucial, as local and incremental expansion in range will ultimately determine the extent of the economic, social and environmental impact of an NNS (Ashton et al. 2006b; Forrest et al. 2009).

Another emerging dimension of NNS spread is through the rapid expansion of artificial, novel habitats, which are expanding in scope, scale and distribution throughout a diversity of intertidal and subtidal marine settings, as a result of increased coastal populations, trade, tourism and the exploitation of natural resources (Mineur et al. 2012). In Europe, it has been estimated that 22,000 km^2^ of the coastal zone has been ‘hardened’ by artificial surfaces, and in some areas more than 50 % of the coastline has been modified (Airoldi and Beck 2007; Dafforn et al. 2015a). NNS are highly opportunistic and are more likely to occur on these novel substrates than on adjacent natural surfaces compared with native species (Connell 2001; Dafforn et al. 2012; Airoldi et al. 2015). Artificial structures are particularly prevalent in harbours and marinas (Rivero et al. 2013), and these locations are subjected to high propagule pressure from NNS due to the volume of traffic concentrated in a specific area and the high occurrence of NNS in ballast water, sea chests and on vessel hulls (Clark and Johnston 2009; Mineur et al. 2012).

The concept of ecological engineering has emerged in recent years as an attempt to integrate ‘ecological, economic and social needs into the design of man-made ecosystems’ (Firth et al. 2014), addressing the concurrent but competing drivers of essential coastal infrastructure and need for habitat restoration (Dafforn et al. 2015b). There have been a number of studies which attempt to alter the design of artificial structures to enhance native biodiversity (Dafforn et al. 2015a; Firth et al. 2014). However, there is a pressing need to more specifically design infrastructure to prevent the establishment of NNS. Similar to the growing trend of environmentally responsible ‘green build’ structures on land, developers could embrace similar design and build concepts into coastal marine infrastructure (Dafforn et al. 2015b), incorporating biosecurity considerations which could potentially reduce NNS establishment.

To reduce the likelihood of NNS occurrence in marinas, there is a need for research to identify the relationships between the physical features of marina infrastructure and the presence of NNS (Airoldi et al. 2015). Additionally, this information needs to be integrated into policy and practices related to coastal marine infrastructure development (Wilson et al. 2015). This study aims to identify specific features of UK marinas which might influence the establishment of NNS, and draws on information from marina operators and users to provide recommendations for a more holistic approach to biosecurity across the recreational boating industry.

## Methods

### Identifying marinas with non-native species

The contact details for 213 marinas and harbours around the UK were compiled from the Practical Boat Owners online marina guide (Practical Boat Owner 2013). This was expanded to 239 marinas using information from data sets listed below.

A list of brackish and marine NNS recorded in UK marine or coastal habitats was compiled based on published reviews (Eno et al. 1997; Arenas et al. 2006; Minchin et al. 2013), literature searches and unpublished field studies. Non-native fish species and microorganisms were excluded from the list. A total of eight existing inventories with information on the presence and/or absence of NNS in one or more of the 239 marinas were reviewed and the data for each marina compiled. These included published inventories and papers by Beveridge et al. (2011), Holt and Cordingley (2011), Arenas et al. (2006) and Ashton et al. (2006a), Minchin and Nunn (2013), Nall et al. (2015), and unpublished records from Scottish marina surveys conducted by Cook (2013, unpubl.). Any species identified by these data sets were then searched for using the NBN Gateway (National Biodiversity Network 2011), in order to locate additional survey records.

### Marina location and construction information

The location and construction details of the marinas, identified as having data on NNS presence, were then investigated with virtual site surveys using the ‘Path’ measuring tool in Google Earth (version 7.1) (Google 2013). The accuracy of aerial and satellite images, which were taken before 2008, was checked with marina websites and online marina guides where available.

Measurements and details were taken of six specific marina features; (i) entrance width (m) at the narrowest point between structures that enclosed the marina. Marina entrances were grouped into three categories; (1) <30 m (enclosed), (2) >30 m (semi-enclosed) and (3) not surrounded by structures (open). (ii) Distance (m) to a freshwater source from the mouth of the marina entrance for enclosed or semi-enclosed marinas and from the mid-point of the marina pontoons for open marinas. A freshwater source was defined as any river or stream identifiable on Google Earth and did not take account of size or flow or include other possible sources, such as storm drains. The measurements were grouped into <20, 20–1000 and >1000 m from a freshwater source. (iii) Total pontoon length (m) was measured for each marina and classified as either <450, 450–1100 or >1100 m. Finger pontoons allowing access to individual vessels were excluded from this for ease of measurement. This exclusion results in underestimations of total pontoon length for some marinas, especially large marinas such as Bangor, Co. Down, where there are a high number of secondary finger pontoons. (iv) Total length (m) of seawalls, vertical structures made of concrete or stone within the marina, was measured and classified as <200, 200–550 or >550 m. (v) Total length (m) of all man-made sloping, boulder structures (i.e. rubble breakwaters) within the marina confines, was measured and classified as either <25, 25–250 or >250 m. (vi) The presence or absence of swing moorings in the marinas was recorded. When marinas had no easily defined boundaries, then moorings were considered to be within the marina vicinity, if they were within 100 m of a pontoon.

### Statistical analysis

A generalised linear model (GLM) with a quasi-Poisson distribution (data were over-dispersed, un-banded count data with a poisson dispersion factor of 2.38) and a log link function was used to analyse the relationship between marina features and NNS richness. Continuous covariates were grouped into classes to prevent outlier influence. Variance inflation factor (VIF) values confirmed that there was no collinearity among these (Zuur et al. 2010). An automated variable selection technique was used, and the relative quality of the models was compared using the quasi-Akaike information criterion (QAIC). The model with the lowest QAIC was considered the best quality, containing only the covariates that explained the variation in NNS counts. The deviance was used to assess the final model fit.

To identify the marina features associated with the presence of each of the five most common NNS (those that occurred in the most marinas), a GLM with a binomial distribution and a logit link function was constructed. Covariates were the same as the quasi-Poisson model described above, with VIF values used to assess collinearity among covariates. Binomial model selection used stepwise automated variable selection with the relative quality of the model assessed using the Akaike information criterion (AIC). The model fit was assessed using the *R*^2^ value to assess to what extent the model could explain the variance of the data.

All data were analysed using the software R version 3.0.1 (R Core Team 2013) in the integrated development environment RStudio, version 0.97.551 (RStudio Team 2012). The models were fitted using the function ‘GLM’ with model selection completed using the ‘dredge’ function in the R package MuMIn for quasi-Poisson models (Barton 2013), and the function ‘step’ for binary models (RStudio Team 2012). The ‘vif’ function in the R package ‘car’ was used to calculate the VIF values for all models (Fox and Weisberg 2011).

### Marina operator and recreational sailor surveys

A survey designed to gather further information on marina design and marina management practices was sent to all marinas and harbours on the contact list compiled from the Practical Boat Owners marina guide (Practical Boat Owner 2013). Marina operators were asked a series of questions on marina use, dominant construction materials and fouling species, and on best management practices (BMP) for cleaning of submerged structures. Recreational sailors were invited to participate in a separate, online survey designed to investigate boating practices and attitudes towards hull-cleaning practices. The recreational sailor survey was disseminated via a public internet forum, the Yachting and Boating World forum (Yachting and Boating World 2013), and on the Practical Boat Owners online news feed (Hodgetts 2013). Both surveys were electronic, constructed using SurveyMonkey (2013) and included both multiple choice and open-ended questions (see Online Resource 1). Both surveys were left open for a period of 42 days during June and July 2013. Responses were tabulated and answers to open-ended questions grouped into themes for analysis.

## Results

### Non-native species in UK marinas

**Fig. 1:**
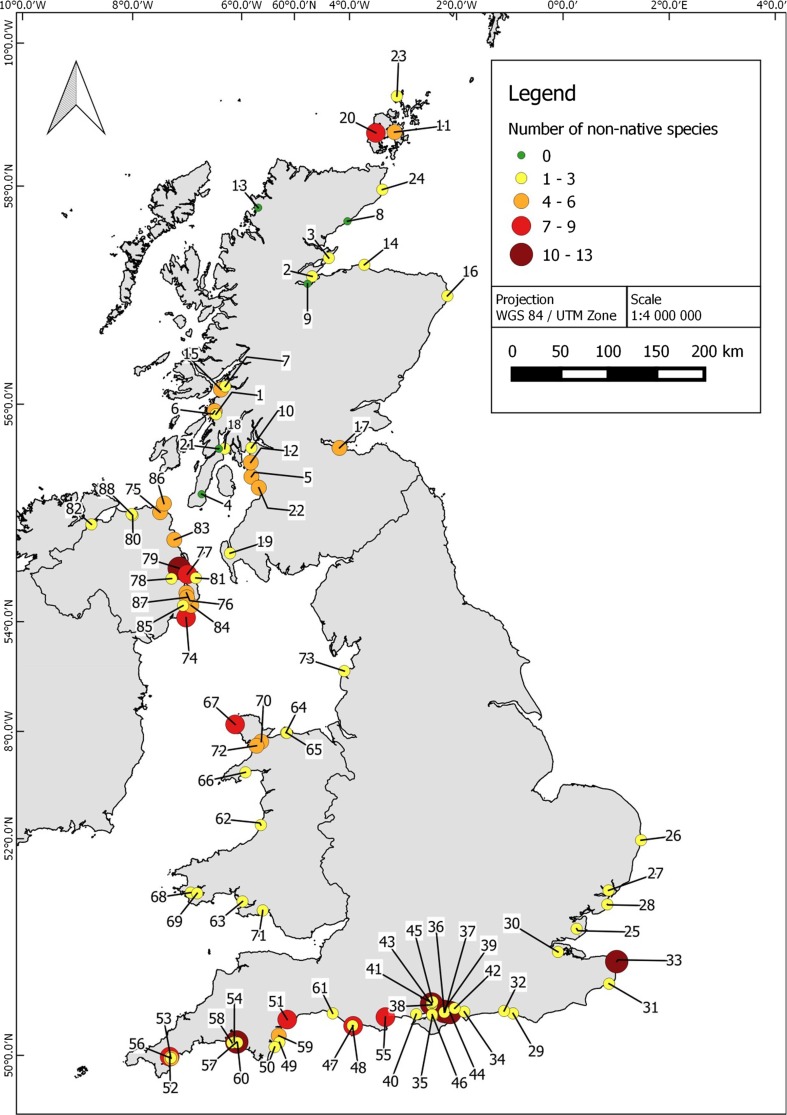
Map of the UK showing distribution of marinas which have been surveyed for non-native species. The total number of different non-native species is represented by the size and *colour of the circle*. The reference numbers for each marina are in Online Resource 3

A review of brackish and marine NNS recorded in UK marine or coastal habitats resulted in a species list of 105 species (Online Resource 2). This list includes species for which there is only a single record, and which may not be established in the UK. A discussion of the criteria for classification of species as non-native and established is provided in Minchin et al. (2013) for the majority of species listed. Of these, 105 NNS known to occur in UK waters, 31 % of these species (*n* = 33) were found to occur in UK marinas. Of the 239 UK marinas identified in our analysis in July 2013, only 88 (35 %) had data on the presence/absence of NNS (Fig. 1).

The five most commonly occurring species in the UK marinas surveyed were the barnacle *Austromininus modesus*, the ascidians *Styela clava* and *Corella eumyota*, the bryozoan *Tricellaria inopinata* and the macroalga *Undaria pinnatifida*. An additional six species, which were included in rapid assessment survey target lists (*Asparagopsis armata*, *Anotrichium furcellatum*, *Bonnemaisonia hamifera*, *Crassostrea gigas*, *Diadumene lineata* and *Eriochier sinensis*), were reported as absent from the marinas surveyed for them (Table 1).

**Table 1:** Non-native species for which marina surveys have been conducted, including the number of marinas each species occurred in by region, as well as the total number of marinas across the UK from which each species was recorded

Phylum	Species	Scotland	Wales	England	Northern Ireland	UK total
Annelida	*Ficopomatus enigmaticus*	0	2	3	0	5
	*Hydroides ezoensis*	0	0	1	0	1
Arthropoda	*Amphibalanus improvisus*	0	0	0	1	1
	***Austrominius modestus***	**7**	**9**	**11**	**8**	**35**
	*Caprella mutica*	12	0	1	6	19
	*Eriocheir sinensis*	0	0	0	0	0
	*Gammarus tigrinus*	0	1	1	2	4
	*Monocorophium acherusicum*	0	0	0	1	1
	*Monocorophium sextonae*	0	0	0	2	2
	*Monocorophium insidiosum*	0	0	0	6	6
Bryozoa	*Bugulina fulva*	0	0	0	2	2
	*Bugula neritina*	0	2	11	1	14
	*Bugulina simplex*	1	0	2	5	8
	*Schizoporella japonica*	3	0	0	0	3
	***Tricellaria inopinata***	**11**	**0**	**9**	**5**	**25**
	*Watersipora subtorquata*	0	0	0	1	1
Chordata	*Aplidium glabrum*	0	0	1	3	4
	*Botrylloides violaceus*	2	0	5	1	8
	***Corella eumyota***	**4**	**3**	**5**	**10**	**22**
	*Didemnum vexillum*	1	0	0	1	2
	*Perophora japonica*	0	0	2	0	2
	***Styela clava***	**2**	**4**	**23**	**3**	**32**
Cnidaria	*Cordylophora caspia*	0	0	0	3	3
	*Diadumene lineata*	0	0	0	0	0
Mollusca	*Crassostrea gigas*	0	0	0	0	0
	*Crepidula fornicata*	0	0	8	0	8
	*Potamopyrgus antipodarum*	0	0	0	1	1
Chlorophyta	*Codium fragile* ssp. *fragile*	8	0	7	0	15
Ochrophyta	*Colpomenia peregrina*	0	1	1	5	7
	*Sargassum muticum*	3	2	6	4	15
	***Undaria pinnatifida***	**0**	**0**	**22**	**1**	**23**
Rhodophyta	*Anotrichium furcellatum*	0	0	0	0	0
	*Asparagopsis armata*	0	0	0	0	0
	*Bonnemaisonia hamifera*	0	0	0	0	0
	*Grateloupia subpectinata*	0	0	2	0	2
	*Grateloupia turuturu*	0	0	6	0	6
	*Dasysiphonia japonica*	4	0	0	0	4
	*Neosiphonia harveyi*	0	0	9	0	9
	*Solieria chordalis*	0	0	1	0	1
Total number of species		12	8	22	22	33

The five most common species are highlighted in bold

**Fig. 2:**
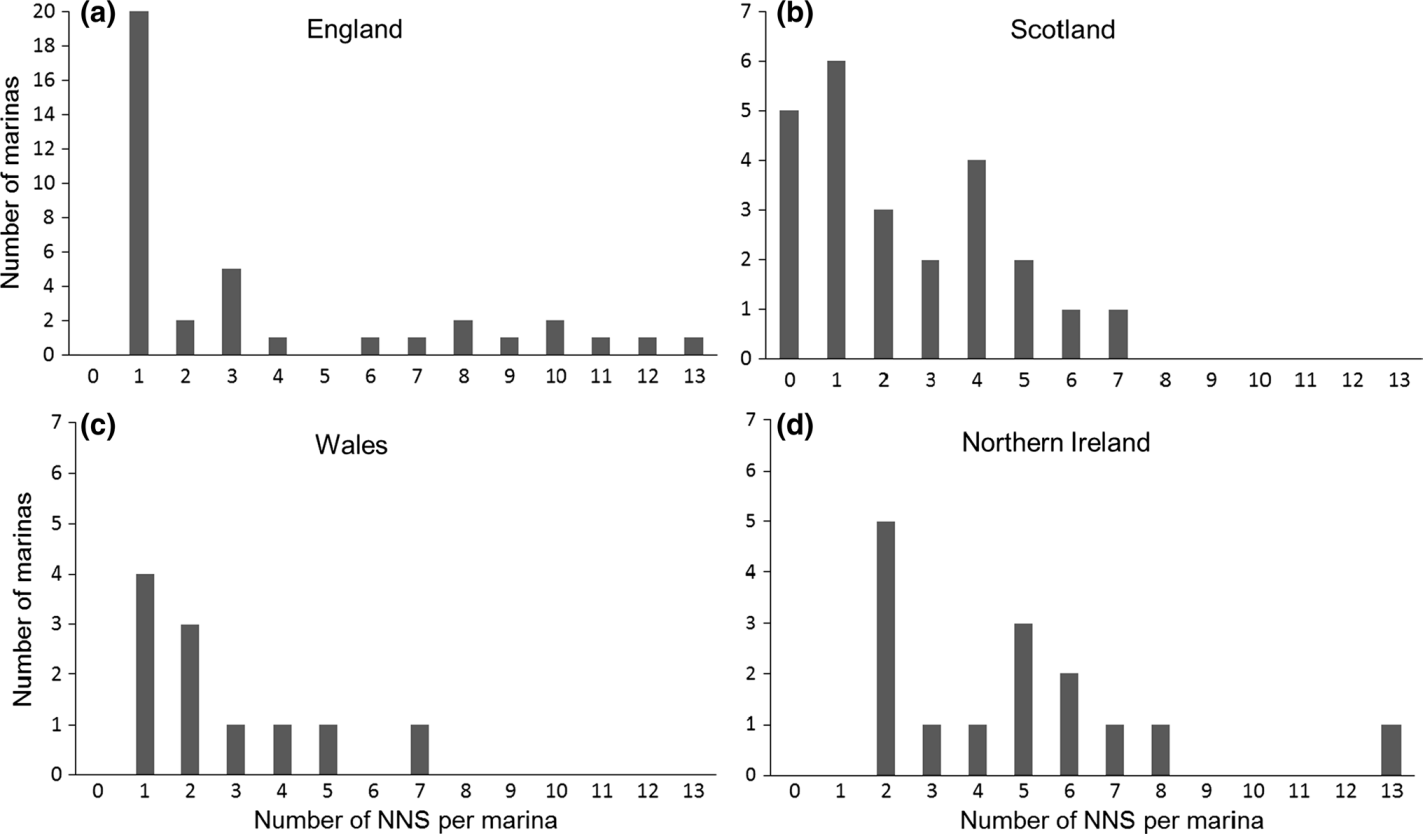
*Bar charts* showing frequency distribution of marina NNS counts for each region of the UK. *Each graph* shows the number of marinas in which NNS surveys were carried out plotted against the number of NNS detected per marina. **a** England (*n* = 38), **b** Scotland (*n* = 24), **c** Wales (*n* = 11) and **d** Northern Ireland (*n* = 15). Note that scales along the *y* axis are not the same for each region. Scotland is the only region with documented absences of NNS (*n* = 5) where NNS were not found during targeted surveys

Of the 88 marinas with data on NNS, the maximum number of NNS recorded in a single marina was 13. Marinas on the south coast of England and in Northern Ireland typically had the greatest number of NNS. Over 92 % of marinas with NNS data had between 1 and 8 NNS (Fig. 2), and 49 % of the marinas contained 1–2 NNS. Scotland and Wales had no more than seven different NNS reported in one marina, while the majority of marinas in these areas contained five or less NNS (Fig. 1). Only five marinas (6 %) surveyed for NNS were not found to have any of the listed NNS species present.

### Marina features and non-native species

**Fig. 3:**
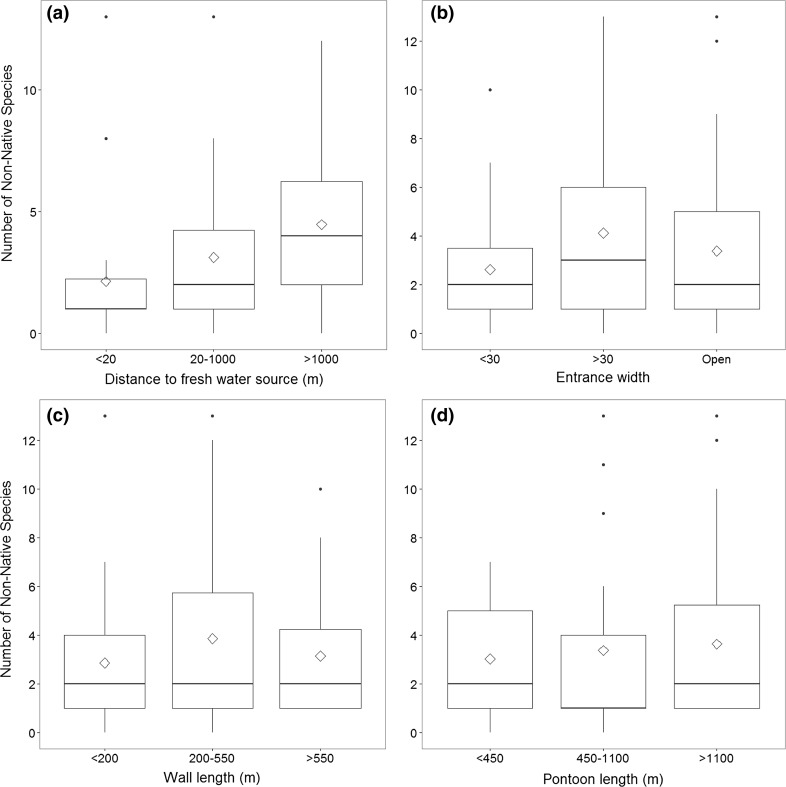
*Box plot* showing of non-native species per marina in relation to marina features retained in GLM model. **a** Distance of marina from a fresh water source [<20 m (*n* = 28), 20–1000 m (*n* = 24), >1000 m (*n* = 36)]. **b** Marina entrance width [<30 m (*n* = 27), >30 m (*n* = 25) or open (*n* = 36)]. **c** Seawall length [<200 m (*n* = 30), 200–550 m (*n* = 20)]. **d** Pontoon length [<450 m (*n* = 31), 450–1100 m (*n* = 21), >1100 (*n* = 36)]. *Box plots* show median values (*solid horizontal line*), mean values (*open diamonds*), 50th percentile values (*box outline*), ±1.5 of the interquartile values (*whiskers*) and outlier values (*black circles*)

Four out of the six marina features were included in the final fitted model for NNS counts (Table 2). Moorings and breakwaters were the two features excluded from this model, as neither explained any of the variation in the NNS counts. The probability of NNS presence in a marina increased significantly (*α* = 0.05) as the distance from a freshwater source increased, *β* = 0.90, *p* < 0.001 (Fig. 3a). The probability of a semi-enclosed marina containing NNS was significantly higher than an open marina or an enclosed marina, *β* = 0.60, *p* < 0.05 (Fig. 3b). There was also a significantly greater probability of a marina containing NNS with 200–550 m of seawalls compared with those with <200 or >550 m, *β* = 0.49, *p* < 0.05 (Fig. 3c). Pontoon length was not significant in predicting the number of NNS in a marina, but it appeared to explain some of the variance in the data and so was retained in the model (Fig. 3d). This model was considered a ‘good fit’ and explains a significant amount of variability in the data, as indicated by the deviance, Model *χ*^2^(8) = 56.44, *p* < 0.001.

**Table 2:** GLM output for the non-native species count with key marina features as covariables

Coefficients	*β* (SE)	95 % CI for odds ratio		
		Lower	Odds ratio	Upper
Intercept	−0.09 (0.35)			
Fresh 20–1000	0.54 (0.27)	1.00	1.71	2.94
Fresh >1000	0.90 (0.24)***	1.56	2.46	3.97
Entrance >30	0.60 (0.25)*	1.13	1.83	2.99
Entrance open	0.27 (0.24)	0.83	1.31	2.13
Seawall 200–550	0.49 (0.21)*	1.08	1.63	2.51
Seawall >550	0.07 (0.27)	0.63	1.07	1.80
Pontoon 450–1100	0.07 (0.25)	0.64	1.07	1.76
Pontoon >1100	0.41 (0.23)	0.97	1.51	2.36

Includes beta (*β*), standard error (SE), odds ratios and upper and lower 95 % confidence intervals (CI) for odds ratio for each coefficient

Model *χ* ^2^(8) = 56.44, *p* < 0.001

Significance codes: *** 0.001; ** 0.01; * 0.05

### Influence of marina features on commonly occurring non-native species

The final binomial GLM models for the five most commonly occurring NNS indicated that different marina features are important for determining whether certain species will be present or absent (Table 3).

**Table 3:** Outputs of binomial GLMs for each of the five most common non-native species, with rank indicating the most common species (1–5), coefficients of the key marina features, beta (*β*) and standard error (SE)

Species	Rank	Coefficients	*β* (SE)	*z* value	*R* ^2^
*A. modestus*	1	Intercept	0.51 (0.28)	1.85	0
*S. clava*	2	Intercept	−3.58 (1.14)**	−3.13	0.53
		Seawall 200–550	2.91 (1.04)**	2.81	
		Seawall >550	3.31 (1.17)**	2.82	
		Pontoon 450–1100	−0.21 (0.96)	−0.22	
		Pontoon >1100	2.87 (1.11)**	2.59	
		Entrance >30	1.38 (0.96)	1.44	
		Entrance open	2.24 (1.11)*	2.02	
*T. inopinata*	3	Intercept	−1.81 (1.64)	−1.11	0.65
		Freshwater 20–1000	−0.09 (1.54)	−0.06	
		Freshwater >1000	3.22 (1.75)	1.84	
		Breakwater 25–250	−4.39 (1.74)*	−2.52	
		Breakwater >250	−0.97 (1.68)	−0.58	
		Mooring	3.62 (1.93)	1.89	
		Entrance >30	4.25 (1.84)*	2.30	
		Entrance open	2.63 (1.61)	1.64	
*U. pinnatifida*	4	Intercept	−17.57 (2284.10)	−0.01	0.58
		Breakwater 25–250	0.97 (1.48)	0.65	
		Breakwater >250	−1.89 (1.06)	−1.78	
		Pontoon 450–1100	17.59 (2284.10)	0.01	
		Pontoon >1100	19.86 (2284.10)	0.01	
*C. eumyota*	5	Intercept	1.12 (0.58)	1.94	0.25
		Breakwater 25–250	−1.73 (0.85)*	−2.04	
		Breakwater >250	−0.27 (0.88)	−0.31	
		Pontoon 450–1100	−2.18 (1.01)*	−2.16	
		Pontoon >1100	−0.65 (0.79)	−0.83	

Significance codes: *** 0.001; ** 0.01; * 0.05

The ascidian *S. clava* occurred in 13 % of the marinas (*n* = 32). The final model for this species found that there was a highly significant probability of *S. clava* being present in a marina as seawall length increased (*β* = 3.31, *p* < 0.01), total pontoon length exceeded 1100 m (*β* = 2.87, *p* < 0.01) and where marinas were classified as ‘open’ compared with ‘enclosed’ (*β* = 2.24, *p* < 0.05). The final model explained 53 % of the variability in the presence of *S. clava* (*R*^2^ = 0.53).

*Tricellaria inopinata* was found in 11 % marinas (*n* = 25). There was a significantly greater probability of *T. inopinata* being present in semi-enclosed compared with enclosed marinas (*β* = 4.25, *p* < 0.05). There was a significantly greater probability of this species being present in marinas where the boulder breakwaters were 25 m or less compared with between 25 and 250 m long (*β* = −4.39, *p* < 0.05). Although the presence of moorings and the distance of the marina from freshwater were not significant, they were retained as they explained some of the variance in the data. The full model explained 65 % of the variability in the presence of *T. inopinata* (*R*^2^ = 0.65).

*Corella eumyota* occurred in 9 % UK marinas (*n* = 22). There is a significantly greater probability of *C. eumyota* being present when the boulder breakwaters are <25 m compared with between 25 and 250 m long (*β* = −1.73, *p* < 0.05). The probability of *C. eumyota* being present is also significantly greater when the pontoon length is <450 m compared with between 450 and 1100 m (*β* = −2.18, *p* < 0.05). The full model only explains 25 % of the variability in the presence of *C. eumyota* (*R*^2^ = 0.25).

In the case of the barnacle *Austrominius modestus*, which was ranked as most common NNS, occurring in 15 % of the marinas (*n* = 35) and the Japanese kelp *U. pinnatifida* which was recorded in 10 % of the marinas (*n* = 23), it appears that none of the features can be used as indicators for the presence of these species.

### Marina operator survey

Of the 213 marinas contacted, 40 marina operators completed the survey in full (18.8 % response rate). The majority of the boat traffic occurring in 93 % of the marinas was recorded as recreational, with only three marinas reporting that both recreational and commercial traffic were equally prevalent. Ninety-five per cent of the marinas reported that their traffic was primarily from the UK, with only two reports of European traffic and one of worldwide traffic (beyond UK and European waters).

Marina operators were asked whether they were aware of NNS within their marina. Twenty-five per cent of marina operators stated that they did have NNS present (*n* = 10), 32.5 % stated they did not (*n* = 13), and 42.5 % were unsure (*n* = 17). Of all 40 of the respondents, 18 marinas were known to contain NNS; however, only three of these responded that they were aware of NNS in their marina, while nine stated they were unsure. Six marinas stated they were not aware of any NNS in their marinas, including one in which nine NNS have been recorded.

Thirty-nine respondents gave information regarding BMP employed for cleaning structures below the water line. Twenty-six per cent of the respondents confirmed they had BMPs for structure cleaning, while the majority did not (54 %). Twenty-one per cent of marina operators who responded to the questionnaire were unsure whether they had BMPs. Of the marina operators that did have BMPs for cleaning underwater structures, e.g. floats, pontoons and slipways, 30 % scraped off fouling organisms, 10 % dry docked pontoons, and 10 % washed down pontoons periodically. Forty per cent of respondents with BMPs stated that they used filters or similar devices to collect and dispose of debris from their onshore boat-washing facilities.

### Recreational sailor survey

There were 105 responses to the online survey, of which 100 were fully completed and used in subsequent analysis. All responses were from recreational sailors in UK waters, with ten respondents also sailing on a commercial basis.

Eighty-nine respondents owned their own vessels. Eighty per cent of owners sailed most frequently within UK waters, 16 % travelled primarily within European waters and 4 % travelled worldwide. Over 50 % of boat owners applied antifouling as frequently as required by the paint manufacturer with no other fouling removal in between applications. Thirty-eight per cent additionally scrubbed vessel hulls to remove fouling between antifouling paint applications. Fewer than 8 % of owners did not use antifouling paint on the hulls of their boats. During the spring and summer sailing season, most owners sailed their boat regularly each month (62 %), while 10 % sailed less than once per month. The average residency time in a marina, other than the home mooring, was commonly 24 h–3 days.

Ninety per cent of respondents stated they knew about NNS. Participants were then asked whether they would be more likely to store their boat at a marina with ‘green credentials’ demonstrating that the marina actively participated in controlling the introduction and growth of NNS. Forty-one per cent of respondents stated that they probably, or definitely, would. Respondents were also asked their opinion about being encouraged, or being required by law to clean the hull of their boat before leaving a UK marina known to be a hot spot for NNS. There were 93 responses to this question, and answers were classified as falling into three categories: (1) against, (2) undecided or (3) supportive. Sixty-three per cent of respondents were against the hull cleaning, with the majority stating that the main reason would be the associated costs of hauling out and removing fouling. Other reasons cited for opposition to mandatory hull cleaning included the addition time it would require, the lack of facilities and the impractical nature of doing this numerous times a year. Ten per cent of respondents also raised the point that recreational boaters should not be penalised for a problem that they believe to be primarily created by the commercial shipping industry. Of the 26 % of respondents who supported the concept, over 25 % raised concerns about the cost and time required for hull cleaning.

## Discussion

### Non-native species records in UK marinas

This study identified 33 NNS recorded in UK marinas. A high proportion of UK marinas where surveys had been conducted had one or more NNS (94 %), and 45 % had three or more. This aligns with other studies which suggest that man-made artificial environments within marinas are highly suitable for the establishment of NNS (Ruiz et al. 2000; Airoldi et al. 2015; Dafforn et al. 2015a). Of the 105 NNS recorded in UK waters in 2013, 31 % were recorded in marinas. A similar percentage of known NNS (34 %) was recorded by Pederson et al. (2003) for 20 marinas along the north-eastern coast of the USA. Data on the abundance of NNS and native species community composition in each marina were not available for analysis in this study, thus making it hard to detect any impact by the NNS; however, studies have previously found that introduced and cryptogenic species are frequently more abundant than native species in marinas and harbours (López-Legentil et al. 2015).

Overall, the number of NNS per marina varied between regions of the UK, with marinas located on the south coast of England typically having the greatest number of NNS (Fig. 1). Initial records of new NNS are often from sites in the English Channel, reflecting the high volume of international and recreational traffic and proximity to European sites compared to elsewhere in the UK (Minchin et al. 2013). Lower sea-surface temperature, current patterns and less vessel activity may be factors in explaining the lower numbers of NNS in Scottish marinas relative to high numbers of NNS found elsewhere in the UK (Nall et al. 2015). The majority of recreational vessel movement is within UK waters, with 80 % of vessel owners predominantly travelling within the UK. Nevertheless, vessels do not need to have arrived from different biogeographic regions to facilitate movement of NNS. Studies have shown recreational vessels can act vectors for the secondary spread of NNS within a region and can connect highly invaded systems with smaller marinas (Minchin 2006; Zabin et al. 2014).

Of the 239 marinas listed in the UK, only 37 % had been surveyed for NNS. Survey effort has not been evenly distributed across the UK, with less than a quarter of English marinas surveyed, compared with over 66 % for Scotland, and 85 and 94 % for Wales and Ireland, respectively. This may, in part, be explained by the higher number of marinas in England. However, there remain areas which have a paucity of data as the majority of studies focus on the south coast, although more recent surveys have extended into under-surveyed areas along the east coast of England (Bishop et al. 2015). The patchiness of survey effort, and the high numbers of NNS present in all surveyed marinas, underpins the need for coordinated, regular long-term monitoring of harbours and marinas across the UK. A potential catalyst is the EU Marine Strategy Framework Directive, which requires the development of a monitoring programme to address the targets developed for NNS, although currently this is still under review in the UK (DEFRA 2014).

The data used in the present study most likely underestimate the presence and distribution of NNS in UK marinas. Many of the original surveys collated in the present study used targeted rapid assessment surveys (RAS) to search for NNS in marinas. RAS are limited to a survey of the top 0.5 m of the water column, missing species which favour deeper water and soft substrates (Ashton et al. 2006a). However, the use of targeted RAS for NNS monitoring has been shown to be time and cost effective in comparison with alternative methods such as extensive baseline surveys or analysis of photograph and scrape samples (Cook et al. 2015). Future surveys of marinas and harbours should attempt to supplement RAS with periodic surveys of the water column and sea floor. Regular monitoring, particularly at ‘hot spot’ sites for NNS establishment, will enable early detection of NNS, increasing the likelihood of a successful control programme.

### Physical features affecting NNS establishment

Marinas located within 20 m of a freshwater source had significantly fewer NNS than those sited over 1 km away. Similar patterns of reduced NNS numbers in marinas with high freshwater input have been observed in the north of Scotland (Nall et al. 2015) and in the USA (Ruiz et al. 2009). Certain NNS have a broad tolerance to variation in temperature and salinity, and there are a number of brackish NNS (Paavola et al. 2005; Gollasch 2006). However, few species will be capable of surviving the transition from fully marine conditions during transfer on hulls of recreational vessels to very low salinity in marinas with a high volume of freshwater (Boos et al. 2011; David et al. 2013). Proximity to a freshwater source was used as an indirect measure of salinity in this study. It is clear that variation in the salinity of the water column may occur in each marina dependent on rainfall and tidal conditions (Bax et al. 2002). Further research, therefore, is required to assess the specific salinity patterns over an extended period in individual marinas to help validate the results of this study. Construction of new marinas in areas of high freshwater input, such as rivers or river mouths, however, would reduce the likelihood of establishment of marine NNS, although care would still be needed to minimise the introduction of brackish and/or freshwater species from other pathways (Kelly et al. 2013).

The results also found that marinas with a medium length of seawall and those with a semi-enclosed entrance had significantly more NNS. Similar trends have been observed for fouling species in other regions, with higher recruitment of species in partially enclosed compared with open marinas (Floerl and Inglis 2003). Enclosed and partially enclosed marinas have complex circulation patterns, which can result in higher water residency and limited dispersal of planktonic larvae, effectively increasing propagule pressure and the likelihood of settlement of NNS larvae (Floerl and Inglis 2003). Reduced tidal flushing also prevents the dispersal of pollutants and sediment, lowering water quality and increasing physical and chemical disturbance of fouling communities (Clark and Johnston 2005; Rivero et al. 2013). Water quality in fully enclosed marinas may be so low that recruitment success of native and non-native fouling species is reduced.

Pontoon length and seawall length represent the availability of hard infrastructure for colonisation by NNS but are also indirect measures of the size of marinas. Size of a marina and corresponding level of vessel activity have been shown to correspond to the likelihood of NNS arriving (Ricciardi 2006; Floerl et al. 2009; Nall et al. 2015). Responses from marina operators suggested the majority of fouling was observed attached to pontoon floats, in agreement with previous work showing a greater prevalence of NNS on floating as opposed to fixed structures (Dafforn et al. 2009b). Nall et al. (2015) also found a greater number of fouling NNS were found in harbours with floating structures. In the results of the GLM model examining marina features, pontoon length did explain some of the variability in the model but was not a significant factor on its own, possibly as a result of interaction with confounding factors such as vessel activity which may also contribute to likelihood of NNS arrival (Ricciardi 2006; Nall et al. 2015).

The physical features assessed in this study are by no means comprehensive, and since this analysis was carried out, other features influencing NNS establishment have been identified. Additional features of marinas identified as influencing NNS prevalence include: underlying habitat (sandy vs. rocky) (Airoldi et al. 2015), harbour type (recreational vs. fishing or commercial use) (López-Legentil et al. 2015), maintenance regime (Airoldi and Bulleri 2011) and levels of disturbance (Dafforn et al. 2009a; Crooks et al. 2011). Of the marinas used in this study, nearly all (93 %) of marina traffic was recreational, suggesting harbour type is unlikely to be a confounding factor in our analysis. Maintenance of marina infrastructure could constitute a disturbance to fouling communities and has been shown to favour NNS establishment (Airoldi and Bulleri 2011). High levels of disturbance are frequently associated with NNS establishment in both terrestrial and marine systems (Davis et al. 2000; Bulleri and Airoldi 2005; Britton-Simmons and Abbott 2008). Marinas frequently have higher levels of disturbance arising from high levels of vessel activity, changed water circulation and chemistry, and high levels of pollution and freshwater run-off (Arenas et al. 2006; Bax et al. 2002; Rivero et al. 2013). However, accurately characterising levels of disturbance requires much more detailed knowledge of the environmental dynamics and operational practices than was feasible for this study.

In addition, geographic distance between marinas was identified by López-Legentil et al. (2015) as significantly related to differences in ascidian communities in Catalan marinas, but was found to be non-significantly related to similarity between marinas in the USA at 1–200 km scales by Lord et al. (2015). The same study identified sea-surface temperature and proximity to areas with high volumes of cargo shipping as influencing variability in NNS richness at large scales (Lord et al. 2015). A study around San Francisco bay and nearby marinas showed smaller fishing and recreational vessels are important in connecting highly invaded ports with smaller marinas and are capable of transferring NNS between them (Zabin et al. 2014). The proximity of marinas in the present study with the highest numbers of NNS (10+) to areas with international shipping warrants further investigation to determine whether the same is true of UK vessels.

The present study focused specifically on fouling NNS and did not consider the relationship between chosen marina features and the native biofouling community, or interactions between native and NNS. Limitations in the availability of data characterising both native and NNS in each marina meant that this was not feasible for a study of this scale. The features identified in this study as influencing NNS, freshwater input and degree of enclosure, will also affect native species dynamics (Floerl and Inglis 2003; David et al. 2013). Characterisation of both native and NNS within marinas with different environmental and physical features is an important next step and may also allow for analysis of impact of NNS establishment.

The five most common NNS identified were all sessile epibenthic species with a relatively short planktonic phase in their life cycle. Recruitment of taxa for which larvae settle quickly has been found to be increased within marinas, while settlement of species with longer planktonic phases is reduced or absent (Rivero et al. 2013). Marina features most correlated with the presence of individual NNS differed across the five most common NNS. This suggests that individual life history characteristics and environmental tolerances may play an important role in predicting which species will establish at a particular location; however, identifying what these are for each individual species is likely to be a complex and time-consuming process. Instead, further analysis of the features identified in this study and others that affect general patterns of NNS settlement may allow for characterisation of the risk of NNS establishment in different marinas. Supplementary analysis of the features identified here and in other studies would allow for guidance to be developed on the features of marinas that can be manipulated to reduce invasion by marine NNS.

#### Current biosecurity practices and attitudes

Our survey of recreational sailors indicated that knowledge of NNS is not uncommon, with over 90 % stating that they knew about NNS. Previous invasive species eradication programmes worldwide have relied on the assistance and awareness of stakeholders (Bax et al. 2002; Holt and Cordingley 2011). However, participants in the survey constitute only a small subset of the wider recreational boating community and the use of the forum to invite survey participants favoured responses from those actively interested in, or antagonistic to, issues of invasive species in marinas. The response of boat owners to proposed changes in boating behaviour demonstrates that there are still barriers, specifically those of cost and time, to the adoption of biosecurity methods. These need to be overcome if the role of recreational vessels as vectors of secondary spread is to be reduced.

Modifications to the design of existing marinas could aid in increasing uptake of current biosecurity recommendations. Novel solutions to reduce biofouling are being trialled, such as rotating pontoon floats (Holt and Cordingley 2011) and in-water encapsulation devices which could reduce costs of treatment to control NNS (Roche et al. 2015). Meanwhile, incorporation of adequate haul out space and methods to prevent removed biofouling from re-entering the water are some of the basic methods that should be considered in any marina development. Awareness of marina operators on the presence of NNS within their own marinas was low, with few respondents correctly identifying whether or not NNS were present. Additionally, just over a quarter of marina operators who responded to the question stated that they had BMPs for the cleaning of underwater structures. As these structures may represent significant reservoirs of NNS, this demonstrates a need for much clearer guidance for marina operators on biosecurity planning for the marina itself (Payne et al. 2014), not just the vessels that use it.

The development of marinas constitutes a significant change to the near-shore environment (Rivero et al. 2013) and results in a site with high potential for increased establishment of NNS. The high prevalence of NNS found within marinas suggests that incorporating biosecurity measures into marina design and operation might greatly reduce secondary spread of NNS by the recreational sailing sector (Cook et al. 2016). While there may be some associated costs with changes to project development processes and operation, over 40 % of sailors questioned indicated that they would preferentially use marinas which offered increased biosecurity. Furthermore, approaching biosecurity from a more holistic, ecosystem- and site-based perspective, in which marina designers, operators and users are actively engaged in marina design and management processes may help lessen the perception that recreational boaters are being overly tasked with the responsibility of preventing NNS movement.

## Electronic supplementary material

Below is the link to the electronic supplementary material.

Supplementary material 1 (PDF 281 kb)

Supplementary material 2 (PDF 221 kb)

Supplementary material 3 (PDF 174 kb)
