# Supplementary material for: Identifying the physical features of marina infrastructure associated with the presence of non-native species in the UK
Source: Mar Biol. 2016 Jul 25;163:173. doi: 10.1007/s00227-016-2941-8 (PMC4960282; doi:10.1007/s00227-016-2941-8)
Supplement: Supplementary file 1 — Supplementary material 1 (PDF 281 kb) [file 227_2016_2941_MOESM1_ESM.pdf]

Identifying the physical features of marina infrastructure associated with the presence of non-native species in the UK
Marine Biology
Victoria Foster[1], Rebecca J. Giesler[2], A. Meriwether W. Wilson[3]*, Christopher R. Nall[4], Elizabeth J. Cook[5]
*University of Edinburgh; meriwether.wilson@ed.ac.uk

Appendix 1: Recreational Sailors Survey and Marina Operators Survey

Appendix 1.1 Transcript of recreational sailor survey. The operational version of this survey was sent out in an electronic format using Survey Monkey.

Question 1: Do you sail recreationally or commercially or both?
- ☐ Recreationally
- ☐ Commercially (End survey)
- ☐ Both

Question 2: Do you own your own boat?
- ☐ Yes (to Question 4)
- ☐ No

Question 3: If you charter, which harbour or marina do you most often charter from? (to Question 10)

Question 4: What size is your boat?

Question 5: Where is your boat primarily moored?

Question 6: How many times per year do you dry stack (or otherwise haul your boat out of the water)?
- ☐ Less than once per year
- ☐ Once
- ☐ Twice
- ☐ Three times
- ☐ More than three times per year
- ☐ Other (please specify)

Question 7: Do you remove the fouling on the hull between hauling your boat out (scrub below the water line to some degree whilst the boat in still in the water)?
- ☐ Yes
- ☐ No

Question 8: Do you use an antifouling paint?
- ☐ Yes
- ☐ No
- ☐ Unsure

Question 9: How frequently do you sail your boat?
- ☐ Weekly
- ☐ A few times a month
- ☐ Less than once a month
- ☐ Other (please specify)

Question 10: If your boat is moored or chartered in UK waters, where do you most often sail to?
- ☐ UK waters
- ☐ Europe excluding the Mediterranean
- ☐ Europe including the Mediterranean
- ☐ Worldwide
- ☐ Other (please specify)

Question 11: On an average trip, what is the maximum time you would spend at another marina/harbour (other than your normal mooring)?
- ☐ Would not stop at another marina
- ☐ 24 hours
- ☐ 3 days
- ☐ One week
- ☐ One month
- ☐ More

Question 12: Are you aware of what a non-native marine species is?
- ☐ Yes
- ☐ No

Question 13: If there was a harbour or marina that had 'green credentials' showing that it actively combats the introduction and growth of non-native species, would you be more inclined to store your boat there (presuming it is at no extra cost, is conveniently located and has facilities you require)?
- ☐ Definitely Yes
- ☐ Probably Yes
- ☐ Maybe
- ☐ Probably Not
- ☐ Definitely Not

Question 14: How would you feel if cleaning the hull of your boat before leaving a UK harbour or marina known to be a hotspot for invasive non-native species was encouraged in the UK? What if it were required by law?

Appendix 1.2 Transcript of marina operators survey. The operational version of this survey was sent out in an electronic format using Survey Monkey.

Question 1: What is the name of your marina?
Question 2: Approximately when was your marina built?
Question 3: What is the capacity of your marina?
Question 4: What is the largest vessel size you can accommodate?
Question 5: Where does the majority of your traffic come from?
- ☐ UK
- ☐ Europe
- ☐ International (beyond Europe)
- ☐ Other (please specify)

Question 6: Is the majority of your traffic recreational or commercial?
- ☐ Recreational (to Question 8)
- ☐ Commercial
- ☐ Other (please specify)

Question 7: If commercial, what maritime industry?
- ☐ Aquaculture
- ☐ Fishing
- ☐ Cargo transport
- ☐ Passenger transport
- ☐ Other (please specify)

Question 8: Which company designed and built the marina? (if unsure, please move on to the next question)
Question 9: Who now owns and/or manages the marina

Question 10: Is there an Environmental Impact Assessment for the marina? If yes, who conducted it and would it be possible to get a copy?

Question 11: Are there any other documents related to the choice in location and size of your marina, or regarding environmental considerations made when the marina was constructed?

- ☐ Yes
- ☐ No
- ☐ Unsure

Question 12: What is the dominant building material below the water line?

- ☐ Concrete
- ☐ Plastic
- ☐ Wood
- ☐ Other (please specify)

Question 13: Is there a river or other source of freshwater flowing through your marina?

- ☐ Yes
- ☐ No
- ☐ Unsure

Question 14: Describe dominant flora/fauna attached to your marina structures (please see email for links to pictures of these organisms)

- ☐ Brown algae (e.g. Kelp)
- ☐ Red algae
- ☐ Green algae
- ☐ Mussels
- ☐ Barnacles and/or limpets
- ☐ Anemones
- ☐ Other (please specify)

Question 15: Which structures are these organisms attached to?

Question 16: Do you have invasive species in your marina?

- ☐ Yes
- ☐ No (to Question 18)
- ☐ Unsure (to Question 18)

Question 17: If yes, do you know which invasive species are present in your marina? (please list all known, either common or scientific names)

Question 18: Do you have facilities to lift boats clear of the water and wash them down?

- ☐ Yes
- ☐ No (to Question 20)
- ☐ Unsure (to Question 20)
- ☐ Other (please specify)

Question 19: If yes, does your wash down facility treat the waste water and prevent any debris returning into the marina basin?

- ☐ Yes
- ☐ No
- ☐ Unsure

Question 20: Do you have best management practices for cleaning marine structures below the water line at your harbour? If yes please give a brief description

- ☐ Yes (Describe management practices)
- ☐ No
- ☐ Unsure
