# Supplementary material for: Identifying the physical features of marina infrastructure associated with the presence of non-native species in the UK
Source: Mar Biol. 2016 Jul 25;163:173. doi: 10.1007/s00227-016-2941-8 (PMC4960282; doi:10.1007/s00227-016-2941-8)
Supplement: Supplementary file 2 — Supplementary material 2 (PDF 221 kb) [file 227_2016_2941_MOESM2_ESM.pdf]

Identifying the physical features of marina infrastructure associated with the presence of non-native species in the UK
Marine Biology
Victoria Foster[1], Rebecca J. Giesler[2], A. Meriwether W. Wilson[3]*, Christopher R. Nall[4], Elizabeth J. Cook[5]
*University of Edinburgh; meriwether.wilson@ed.ac.uk

Appendix 2: List of marine and brackish non-native species recorded in the UK as of 2013, compiled based on published reviews (Eno et al. 1997; Arenas et al. 2006; Minchin et al. 2013), literature searches and unpublished field studies. Species are ordered by taxonomic groups, based on a similar listing in Minchin et al. (2013). Species recorded in marinas are indicated and coloured grey. Reviews in which the species is referenced are listed.

	Species	Recorded in marina? (Y=Yes)	References
**Ocrophyta, Phaeophyceae**			
	Undaria pinnatifida (Harvey) Suringar, 1872	Y	Eno 1997; Arenas 2006; Minchin 2013; Nunn 2013
	Colpomenia peregrina (Sauvageau) Hamel, 1927	Y	Eno 1997; Arenas 2006; Minchin 2013; Nunn 2013; Sambrook 2014
	Sargassum muticum (Yendo) Fensholt, 1995	Y	Eno 1997; Arenas 2006; Minchin 2013; Nunn 2013; Sambrook 2014
**Rhodophyta, Florideophyceae**			
Bonnemaisoniaceae	*Bonnemaisonia hamifera* Harriot, 1891		Eno 1997; Minchin 2013; Sambrook 2014
Ceramiaceaea	*Antithamnionella spirographidis* (Schiffner) Wollaston, 1968		Eno 1997; Minchin 2013; Sambrook 2014
	Antithamnionella ternifolia (C. Agardh) Nageli, 1847		Eno 1997; Minchin 2013; Sambrook 2014
	Asparagopsis armata Harvey, 1855		Eno 1997; Minchin 2013; Nunn 2013; Sambrook 2014
Caulacanthidae	*Caulacanthus ustulatus* (Mertens ex Turner) Kützing, 1843		Minchin 2013
Dasyaceae	*Dasysiphonia japonica* (Yendo) H.-S.Kim, 2012	Y	Beveridge 2011; Minchin 2013; Nunn 2013; Nall 2013
Dumontiaceae	*Pikea californica* Harvey, 1853		Eno 1997; Minchin 2013
Gigartinaceae	*Chondracanthus acicularis* (Roth) Fredericq 1993		Nunn 2013
Gracilariaceae	*Gracilaria vermiculophylla* (Ohmi) Papenfuss, 1967		Nunn 2013
Halymeniaceae	*Cryptonema Hibernica* Guiry & L. Irvine, 1974		Minchin 2013
	Grateloupia luxurians (subpectinata) Holmes, 1912	Y	Eno 1997; Arenas 2006; Minchin 2013
	Grateloupia turuturu Yamada, 1941	Y	Arenas 2006; Minchin 2013
Rhodomelaceae	*Neosiphonia harveyi* J.W. Bailey, 1848	Y	Eno 1997; Arenas 2006; Minchin 2013; Sambrook 2014; Nall 2013
Solieriaceae	*Sarcodiotheca gaudichaudi* (Montagne) P.W. Gabrielson, 1982		Minchin 2013
	Solieria chordalis (C. Agardh) J. Agardh, 1842	Y	Eno 1997; Arenas 2006; Minchin 2013; Sambrook 2014
	Agardhiella subulata (C.Agardh) Kraft & M.J.Wynne, 1979		Eno 1997
Wrangeliaceae	*Anotrichium furcellatum* (J.Agardh) Baldock, 1976		Arenas 2006

**Chlorophyta, Bryopsidophyta**			
Codiaceae	*Codium fragile subsp. fragile* (Proven, Booth,Todd, Beatty, & Maggs, 2008)	Y	Eno 1997; Arenas 2006; Minchin 2013; Nunn 2013; Nall 2013
	Codium fragile subsp. atlanticum (A.D.Cotton) P.C.Silva, 1955		Eno 1997
**Anthophyta**, Poaceae			
	Spartina townsendii var. anglica C.E. Hubbard		Eno 1997; Minchin 2013
**Cnidaria, Anthozoa**			
Bougainvilliidae	*Pachycordyle navis* (Millard, 1959)		Eno 1997
Cordylophoridae	*Cordylophora caspia* (Pallas, 1771)	Y	Nunn 2003
Diadumenidae	*Diadumene lineata* (Verrill, 1869)		Eno 1997; Minchin 2013; Nunn 2013; Sambrook 2014
Olindiasidae	*Gonionemus vertens* A. Agassiz, 1862		Eno 1997; Minchin 2013
**Nematoda, Dracunculoidea**			
Anguillicolidae	*Anguillicola crassus* Kuwahara, Niimi & Itagaki, 1974		
**Annelida, Polychaeta**			
Goniadidae	*Goniadella gracilis* Verrill, 1873		Eno 1997; Minchin 2013; Sambrook 2014
Maldanidae	*Clymenella torquata* (Leidy, 1855)		Eno 1997; Minchin 2013; Nunn 2013
Sabellidae	*Desdemona ornate* Banse, 1957		Minchin 2013
Serpullidae	*Ficopomatus enigmaticus* (Fauvel, 1923)	Y	Eno 1997; Arenas 2006; Minchin 2013; Sambrook 2014
Serpullidae	*Hydroides dianthus* (Verrill, 1873)		Eno 1997; Minchin 2013
Serpullidae	*Hydroides elegans* (Haswell, 1883)		Minchin 2013
Serpullidae	*Hydroides ezoensis* Okuda, 1934	Y	Eno 1997; Arenas 2006; Minchin 2013
Spionidae	*Marenzelleria viridis* (Verrill, 1973)		Eno 1997; Minchin 2013
Spirorbidae	*Janua brasiliensis* (Grube, 1872)		Eno 1997; Minchin 2013
Spirorbidae	*Pileolaria berkeleyana* (Rioja, 1942)		Eno 1997; Minchin 2013
**Mollusca, Gastropoda**			
Calyptraeidae	*Calyptraea chinensis* Linnaeus, 1758		Minchin 2013; Nunn 2013
Calyptraeidae	*Crepidula fornicata* (Linnaeus, 1758)	Y	Eno 1997; Arenas 2006; Minchin 2013; Nunn 2013; Sambrook 2014
Hydrobiidae	*Potamopyrgus antipodarum* (J.E.Gray, 1843)	Y	Eno 1997; Minchin 2013; Nunn 2013
Muricidae	*Rapana venosa* (Valenciennes, 1846)		Minchin 2013
	Urosalpinx cinerea (Say, 1822)		Eno 1997; Minchin 2013
**Mollusca, Bivalvia**			
Atrinidae	*Atrina rigida* (Lightfoot, 1786)		Minchin 2013
Dreissenidae	*Dreissena polymorpha* (Pallus, 1771)		Minchin 2013
	Mytilopsis leucophaeta Conrad, 1831		Minchin 2013
Myidae	*Mya arenaria* (Linnaeus, 1758)		
Mytilidae	*Aulacomya atra* (Molina, 1782)		Minchin 2013

Mytilidae	*Brachidontes exustus* Linnaeus, 1758		Minchin 2013
	Choromytilus chorus (Molina, 1782)		Minchin 2013
Corbiculidae	*Corbicula fluminea* (O.F. Müller, 1774)		Minchin 2013
Ostreidae	*Crassostrea gigas* (Thunberg, 1793)		Eno 1997; Minchin 2013; Nunn 2013; Sambrook 2014
	Crassostrea rhizophorae Guilding, 1828		Minchin 2013
	Crassostrea virginica (Gmelin,1791)		Minchin 2013; Sambrook 2014
	Dendostrea folium (Linnaeus, 1758)		Minchin 2013
	Ostrea chilensis Philippi, 1844		Eno 1997; Minchin 2013
Petricolidae	*Petricola pholadiformis* Lamark, 1818		Eno 1997; Minchin 2013; Sambrook 2014
Pinctadidae	*Pinctada radiata* (Leach, 1814)		Minchin 2013
Pharidae	*Ensis directus* (Conrad, 1843)		Eno 1997; Minchin 2013
Pteriidae	*Pteria colymbus* (Röding, 1798		Minchin 2013
Teredinidae	*Teredo navalis* (Linnaeus, 1758)		Nunn 2013
Veneridae	*Mercenaria mercenaria* (Linnaeus, 1758)		Eno 1997; Minchin 2013; Sambrook 2014
	Venerupis philippinarum (Adams & Reeve, 1850)		Minchin 2013
**Arthropoda, Maxillopoda, Copepoda, Calanoida**			
Acartiidae	*Acartia tonsa* Dana, 1849		Eno 1997; Minchin 2013
Temoridae	*Eurytemora Americana* L.W. Williams, 1906		Minchin 2013
**Arthropoda, Ostracoda**			
Sarsiellidae	*Eusarsiella zostericola* Cushman, 1906		Eno 1997; Minchin 2013
**Arthropoda, Thecostraca, Cirripedia, Thoracica**			
Austrobalanidae	*Austrominius modestus* Darwin, 1854	Y	Eno 1997; Minchin 2013; Nunn 2013; Sambrook 2014; Nall 2013
Balanidae	*Amphibalanus amphitrite* Darwin, 1854		Eno 1997; Minchin 2013
	Balanus trigonus Darwin, 1854		Minchin 2013
	Amphibalanus improvises Darwin, 1854	Y	Nunn 2013
**Arthropoda, Malacostraca, Amphipoda**			
Aoridae	*Grandidierella japonica* Stephenson, 1938		Minchin 2013
	Gammarus tigrinus Sexton, 1939	Y	Minchin 2013; Nunn 2013
Caprellidae	*Caprella mutica* Schurin, 1935	Y	Arenas 2006; Beveridge 2011; Minchin 2013; Nunn 2013; Nall 2013
Corophiidae	*Monocorophium acherusicum* (Costa, 1853)	Y	Nunn 2013
Corophiidae	*Monocorophium sextonae* (Crawford, 1937)	Y	Eno 1997; Nunn 2013
	Monocorophium insidiosum (Crawford, 1937)	Y	Nunn 2013
**Arthropoda, Malacostraca, Decapoda**			
Nephropidae	*Homarus americanus* (H. Milne Edwards, 1837)		Minchin 2013
Palaemonidae	*Palaemon macrodactylus* Rathbun, 1902		Minchin 2013
Panopeinae	*Dyspanopeus sayi* (S.I. Smith, 1869)		Minchin 2013

Panopeinae	*Rhithropanopeus harrisii* (Gould, 1841)		Eno 1997; Minchin 2013
Penaeidae	*Penaeus japonicas* (Bate, 1888)		Minchin 2013; Nunn 2013
Pilumnoididae	*Pilumnoides perlatus* (Poeppig, 1836)		Minchin 2013
Portunidae	*Callinectes sapidus* Rathbun, 1896		Minchin 2013
Varunidae	*Eriocheir sinensis* (H. Milne Edwards, 1853)		Eno 1997; Minchin 2013; Sambrook 2014
Varunidae	*Brachynotus sexdentatus* (Risso, 1827)		Minchin 2013
**Arthropoda, Chelicerata, Pycnogonida**			
Ammotheidae	*Ammothea hilgendorfi* (Böhm, 1879)		Eno 1997; Minchin 2013
**Bryozoa**			
Bugulidae	*Bugulina fulva (Ryland, 1960)*	Y	Nunn 2003; Nall 2013
	Bugula neritina (Linnaeus, 1758)	Y	Nunn 2003; Arenas 2006; Minchin 2013; Sambrook 2014
	Bugulina simplex (Hinks, 1886)	Y	Nunn 2003; Minchin 2013; Nall 2013
	Bugulina stolonifera (Ryland, 1960)		Minchin 2013; Sambrook 2014
Candidae	*Tricellaria inopinata* d'Hondt & Occhipinti Ambrogi, 1985	Y	Arenas 2006; Cook 2013; Minchin 2013; Nunn 2013; Nall 2013
Schizoporellidae	*Schizoporella japonica* Ortmann, 1890	Y	Nall 2013
Watersiporidae	*Watersipora subtorquata* (d'Orbingny, 1952)	Y	Minchin 2013; Nunn 2013
**Chordata**			
Botryllidae	*Botrylloides diegensis* Oka, 1927		Minchin 2013
	Botrylloides violaceus (Ritter & Forsyth, 1917)	Y	Nunn 2003; Arenas 2006; Minchin 2013; Nall 2013
Corellidae	*Corella eumyota* Traustedt, 1882	Y	Nunn 2003; Arenas 2006; Beveridge 2011; Minchin 2013; Nall 2013
Didemnidae	*Didemnum vexillum* Kott, 2002	Y	Beveridge 2011; Minchin 2013; Nunn 2013
Perophoridae	*Perophora japonica* Oka, 1927	Y	Arenas 2006; Minchin 2013; Sambrook 2014
Polyclinidae	*Aplidium glabrum* (Verrill, 1871)	Y	Nunn 2003
Styelidae	*Asterocarpa humilis* (Heller, 1878)		EJ Cook pers. Comm (2013)
	Styela clava Herdman, 1882	Y	Eno 1997; Arenas 2006; Minchin 2013; Sambrook 2014; Nall 2013
**Cryptogenic species** (These species were not included in our analysis, but were identified by Nall et al. (2015) as being present in marinas in northern Scotland and as such are included here.			
Amphipoda, Ischyroceridae	*Jassa marmorata* Holmes, 1905	Y	Nall 2013

References:

Arenas F, Bishop JDD, Carlton JT, Dyrynda PJ, Farnham WF, Gonzalez DJ, Jacobs MW, Lambert C, Lambert G, Nielsen SE, Pederson JA, Porter JS, Ward S, Wood CA (2006) Alien species and other notable records from a rapid assessment survey of marinas on the south coast of England. J Mar Biol Assoc UK 86: 1329 -1337 doi 10.1017/S0025315406014354

Beveridge C, Cook EJ, Brunner L, MacLeod A, Black K, Brown C, Manson FJ (2011) Initial response to the invasive carpet sea squirt, Didemnum vexillum, in Scotland. Scottish Natural Heritage

Cook EJ, pers comm. (2013) Records from RAS surveys in Scotland.
Eno NC, Clark RA, Sanderson WG (1997) Non-native marine species in British waters: a review and directory. JNCC, Peterbourgh
Minchin D, Cook E, Clark P (2013) Alien species in British brackish and marine waters. Aquat Invasions 8: 3-19 doi 10.3391/ai.2013.8.1.02
Sambrook K, Griffith K, Jenkins SR (2014) Review of monitoring of marine non-native species in Great Britain and evaluation of gaps in data dissemination. NRW Evidence Series. Report No: 20, 36pp, NRW Bangor
Nall CR (2013) Preliminary dataset from rapid assessment surveys of marine non-native species in northern Scotland. A published updated dataset can be found in: Nall CR, Guerin AJ, Cook EJ (2015) Rapid assessment of marine non-native species in northern Scotland and a synthesis of existing Scottish records. Aquatic Invasions 10: 107-121, http://dx.doi.org/10.3391/ai.2015.10.1.11
Nunn J (2003, 2013) Unpublished records of NNS surveys in Northern Ireland. For more information see - Nunn J, Minchin D (2013) Marine non-native invasive species in Northern Ireland. http://invasivespeciesireland.com/wp-content/uploads/2013/03/Julia-Nunn.pdf
