# Supplementary material for: Identifying the physical features of marina infrastructure associated with the presence of non-native species in the UK
Source: Mar Biol. 2016 Jul 25;163:173. doi: 10.1007/s00227-016-2941-8 (PMC4960282; doi:10.1007/s00227-016-2941-8)
Supplement: Supplementary file 3 — Supplementary material 3 (PDF 174 kb) [file 227_2016_2941_MOESM3_ESM.pdf]

Identifying the physical features of marina infrastructure associated with the presence of non-native species in the UK

Marine Biology

Victoria Foster[1], Rebecca J. Giesler[2], A. Meriwether W. Wilson[3]*, Christopher R. Nall[4], Elizabeth J. Cook[5]
*University of Edinburgh; meriwether.wilson@ed.ac.uk

Appendix 3: List of marinas surveyed for non-native species in the UK, ordered by region of the UK. Number of non-native species recorded (No. NNS) has been indicated as well as exact location. Reference numbers relate to Figure 1.

Ref no.	Marina Name	Location	Latitude	Longitude	No. NNS
1	Ardfern Yacht Centre	Scotland	56.1827461	-5.530157738	3
2	Avoch	Scotland	57.56718924	-4.166603389	1
3	Balintore Marina	Scotland	57.75450999	-3.910804169	1
4	Campbeltown Loch	Scotland	55.42448766	-5.601468932	0
5	Clyde Marina	Scotland	55.64169727	-4.821127108	5
6	Craobh Marina	Scotland	56.21123948	-5.557394959	4
7	Dunstaffnage Marina	Scotland	56.45180381	-5.432723251	1
8	Helmsdale	Scotland	58.11466896	-3.648476739	0
9	Inverness Marina	Scotland	57.49376543	-4.233708111	0
10	Kip Marina	Scotland	55.90972815	-4.875667476	1
11	Kirkwall Marina	Scotland	58.98686232	-2.958535946	6
12	Largs Yacht Haven	Scotland	55.77346094	-4.858063156	5
13	Lochinver Harbour	Scotland	58.14828841	-5.245428971	0
14	Lossiemouth Marina	Scotland	57.72367522	-3.280319367	2
15	Oban Marina	Scotland	56.41837473	-5.497237252	4
16	Peterhead Bay Marina	Scotland	57.49645161	-1.791077342	3
17	Port Edgar Marina	Scotland	55.99466065	-3.412386649	4
18	Portavadie Marina	Scotland	55.87352526	-5.314821916	1
19	Stranraer	Scotland	54.90784597	-5.025888389	1
20	Stromness Marina	Scotland	58.96400729	-3.295009648	7
21	Tarbert Harbour	Scotland	55.86623739	-5.411498081	0
22	Troon Yacht Haven	Scotland	55.54735665	-4.678997863	4
23	Westeray Pierowall	Scotland	59.32372014	-2.975257803	2
24	Wick	Scotland	58.44034828	-3.085018108	3
25	Burnham-on-Crouch Harbour	England	51.62779776	0.803970675	1
26	Lowestoft Haven Marina	England	52.47235807	1.752536	3
27	Suffolk Yacht Harbour	England	51.99503951	1.271555933	3
28	Titchmarsh Marina	England	51.86354165	1.257992279	1
29	Brighton Marina	England	50.81122497	-0.100675525	1
30	Chatham Maritime Marina	England	51.40353863	0.535452826	1
31	Dover Marina	England	51.11916943	1.312298432	1
32	Lady Bee Marina	England	50.83084645	-0.234551559	2

33	Ramsgate	England	51.32839239	1.419245821	10
34	Chichester Marina	England	50.80544597	-0.822954692	1
35	East Cowes Marina	England	50.75143966	-1.290534876	1
36	Gosport Marina	England	50.79836352	-1.11962096	12
37	Gunwharf Quays Marina	England	50.79482042	-1.108184069	1
38	Hamble Point Marina	England	50.85271917	-1.309916594	1
39	Haslar Marina	England	50.79062297	-1.118218817	3
40	Lymington Marina	England	50.75700934	-1.53142249	1
41	Mercury Yacht Harbour	England	50.87122539	-1.311276597	1
42	Northney Marina	England	50.83252124	-0.967153761	1
43	Port Hamble Marina	England	50.86106908	-1.312266538	13
44	Southsea Marina	England	50.79126908	-1.035191752	10
45	Swanwick Marina	England	50.88232325	-1.300273547	1
46	West Cowes Marina	England	50.76149073	-1.295363599	1
47	Weymouth Harbour	England	50.60801556	-2.448315585	8
48	Weymouth Marina	England	50.60930406	-2.458065439	3
49	Brixham Marina	England	50.39911513	-3.509380674	3
50	Darthaven Marina	England	50.3506517	-3.571973258	1
51	Exmouth Marina	England	50.61739677	-3.42402188	7
52	Falmouth Marina	England	50.16419387	-5.084296284	8
53	Falmouth Yacht Haven	England	50.15378565	-5.065418115	1
54	Mayflower Marina	England	50.36414333	-4.168678673	1
55	Poole Quay Boat Haven	England	50.71159501	-1.982859203	9
56	Port Pendennis Marina	England	50.15134163	-5.061553629	4
57	Queen Anne's Battery	England	50.36509315	-4.131262312	11
58	Southdown Marina	England	50.35310839	-4.197851908	1
59	Torquay Marina	England	50.45948403	-3.527075673	6
60	Turnchaple Marina	England	50.3590721	-4.121706078	1
61	West Bay Harbour	England	50.71034164	-2.763304598	1
62	Aberystwyth Marina	Wales	52.40877158	-4.087694792	1
63	Burry Port Harbour	Wales	51.67950562	-4.250404369	2
64	Conwy Quays Marina	Wales	53.29193271	-3.839587236	1
65	Deganwy Marina	Wales	53.29105541	-3.82753367	1
66	Hafan Pwllheli	Wales	52.88536686	-4.405740464	2
67	Holyhead Marina	Wales	53.32077209	-4.643612884	7
68	Milford Marina	Wales	51.7114783	-5.039334548	2
69	Neyland Yacht Haven	Wales	51.71206478	-4.941565288	3
70	Port Dinorwic Marina	Wales	53.18589201	-4.210206269	5
71	Swansea Marina	Wales	51.61469348	-3.934482046	1
72	Victoria Dock	Wales	53.14325758	-4.276547081	4
73	Fleetwood Haven Marina	England	53.91617915	-3.012375142	2

74	Ardglass Marina	NI	54.26126868	-5.606060236	7
75	Ballycastle Marina	NI	55.20705961	-6.239214842	4
76	Ballydorn Lightship	NI	54.4910073	-5.649440739	6
77	Bangor Marina	NI	54.66526025	-5.671588842	8
78	Belfast Harbour Marina	NI	54.60607267	-5.915341908	2
79	Carrickfergus Marina	NI	54.71074709	-5.813362629	13
80	Coleraine Marina	NI	55.14441856	-6.676511062	3
81	Copelands Marina	NI	54.6402691	-5.528154845	2
82	Foyle Marina	NI	55.00520037	-7.31985875	2
83	Glenarm Marina	NI	54.96956099	-5.950886345	6
84	Portaferry Marina	NI	54.37922283	-5.548561975	5
85	Quoile Yacht Club	NI	54.36870417	-5.678284263	2
86	Rathlin Harbour	NI	55.29196039	-6.195708747	5
87	Ringhaddy Harbour	NI	54.45003039	-5.632935965	5
88	Seaton's Marina	NI	55.1536986	-6.694874812	2
